# Distal ileal lipoma causing small bowel obstruction without intussusception: A case report

**DOI:** 10.1016/j.ijscr.2025.111588

**Published:** 2025-06-30

**Authors:** Atabak Sedigh-Namin, Mehran Hatabi, Mohammadamin Vatankhah, Meisam Refaei, Elmira Mousavi, Hamed Mohammadi Haris

**Affiliations:** a Students Research Committee, School of Medicine, Ardabil University of Medical Sciences, Ardabil, Iran; b Surgery Department, Ardabil University of Medical Sciences, Ardabil, Iran; c Department of Radiology, Imam Reza Hospital, Tabriz University of Medical Sciences, Tabriz, Iran; d Students Research Committee, School of Medicine, Tabriz University of Medical Sciences, Tabriz, Iran; e Division of Vascular and Endovascular Surgery, Department of Surgery, Shohada-Tajrish Hospital, Shahid Beheshti University of Medical Sciences, Tehran, Iran; f Department of Anatomical Sciences and Pathology, School of Medicine, Dr. Fatemi Hospital, Ardabil University of Medical Sciences, Ardabil, Iran

**Keywords:** Lipoma, Small bowel obstruction, Ileum

## Abstract

**Background:**

Lipomas of the gastrointestinal tract are infrequent, benign neoplasms. They can be found anywhere in the gastrointestinal tract; the ileum is one of the most frequent sites in the small intestine. Intraluminal lipomas of the distal ileum causing bowel obstruction without intussusception are extremely rare.

**Case presentation:**

A 44-year-old male with complaints of abdominal distension, nausea, vomiting, colicky abdominal pain, was subjected to an exploratory laparotomy, which showed intraluminal lipoma situated in the distal ileum causing mechanical obstruction without intussusception. Surgical resection was successfully performed, and the patient recovered with no complications.

**Discussion:**

Small bowel lipomas are usually asymptomatic. However, larger lesions cause a huge symptomatology of bowel obstruction. In the patient studied, the lipoma caused a complete mechanical obstruction of the small bowel without typical complications with intussusception or volvulus. Although gastrointestinal lipomas are relatively infrequent, the case reminds clinicians of the differential diagnosis of the mechanisms of bowel obstruction. While imaging has a critical role in diagnosis, advanced techniques of CT or MRI are more likely to delineate soft tissue lesions further.

**Conclusion:**

This is a case of an intestinal lipoma that presents with a bowel obstruction in the absence of the usual complications such as intussusception or volvulus. Surgical resection remains the preferred treatment for symptomatic and obstructing lipomas, and their consideration in the differential diagnosis is essential for timely management.

## Highlights

• This case reports a rare small bowel obstruction by intraluminal lipoma in the distal ileum without intussusception or volvulus.
• CT or MRI offers better insight into soft tissue lesions like lipomas, helping confirm bowel obstruction suggested by X-rays.
• Surgical resection is preferred for symptomatic obstructing lipomas, especially when causing mechanical obstruction without complications.

## 1 Introduction

This case report follows the SCARE criteria exclusively to ensure a structured and comprehensive presentation of the clinical scenario [1]. Lipomas of the gastrointestinal tract are rare benign neoplasms that can develop anywhere along the tract, although the small intestine is an uncommon location for them [2]. The incidence of gastrointestinal lipomas, as observed in autopsy studies, ranges from 0.035 % to 4.4 %. Most cases are asymptomatic, and there is a slight female predominance, with the highest incidence occurring in individuals during their fifth and sixth decades of life [[3], [4], [5]]. Gastrointestinal lipomas can vary in size, ranging from 2 mm to 30 cm [6]. While the small intestine is an unusual site for lipomas, the ileum is one of the more common sites within it, though intraluminal lipomas in the distal ileum are extremely rare. Most lipomas are asymptomatic, but if they exceed 2 cm, they can lead to significant symptoms, including abdominal pain, nausea, vomiting, and recurring constipation. Although small intestine lipomas are rare, they can conduct to serious complications, particularly when lesions are larger [7]. Symptoms often consist of abdominal pain, and complications such as intussusception may occur, especially if the lipoma is located in the mesentery. While obstruction due to small intestinal lipomas is rare, it can manifest as intussusception or, less commonly, as complete obstruction without intussusception [6]. Other potential complications include peritonitis, perforation, and massive bleeding that require surgical intervention. There are cases of large intraluminal lipomas that obstruct the small intestine, which often requires surgical interventions, such as small bowel resection through laparotomy. We report a unique case of complete small bowel obstruction without intussusception caused by an intraluminal submucosal lipoma of the distal ileum, which has not been previously reported in the literature.

## 2 Case presentation

A 44-year-old male presented to the emergency department with a several-month history of vague, intermittent abdominal pain centered around the umbilicus. He reported a three-day history of abdominal distention, nausea, and vomiting. Over the past 24 h, he had vomited several times, with food and bile contents. The patient also reported an absence of flatus since the previous day. He complained of additional pain around the umbilicus and the lower abdomen. His past medical history was unremarkable, with no prior surgeries or chronic medications. During the examination, he was found to be tachycardic, with a heart rate of 115 beats per minute, and febrile, with a temperature of 38.8 °C. The abdominal examination revealed a soft abdomen with tenderness noted in the lower abdomen, especially in the right lower quadrant (RLQ). Groin examination showed no abnormalities, and a digital rectal exam (DRE) revealed an empty rectum without any abnormal findings. Laboratory results demonstrated leukocytosis (12,000; with neutrophils at 82 %) and mild metabolic acidosis (pH 7.32), while other tests were normal. Following initial stabilization with the insertion of a nasogastric (NG) tube, Foley catheterization, and fluid resuscitation, the patient was taken to the operating room. A midline laparotomy was performed, and exploration revealed a transition zone in the distal ileum, located 20 cm from the ileocecal valve.

**Fig. 1:**
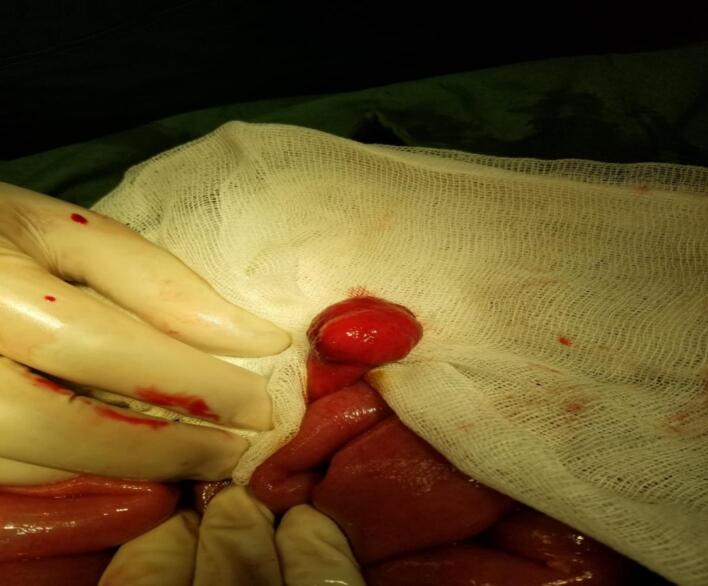
Intraoperative image showing the large pedunculated polyp causing mechanical obstruction.

**Fig. 2:**
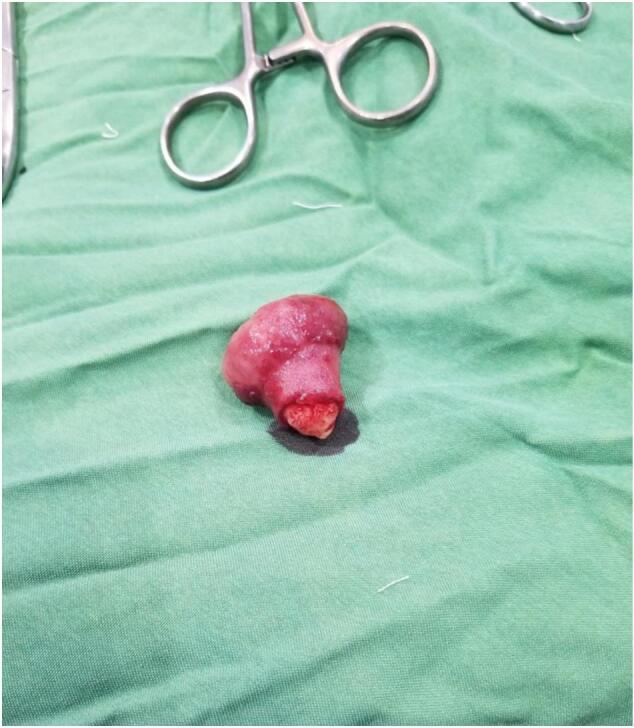
Intraluminal lipoma after excision and its appearance postoperatively.

**Fig. 3:**
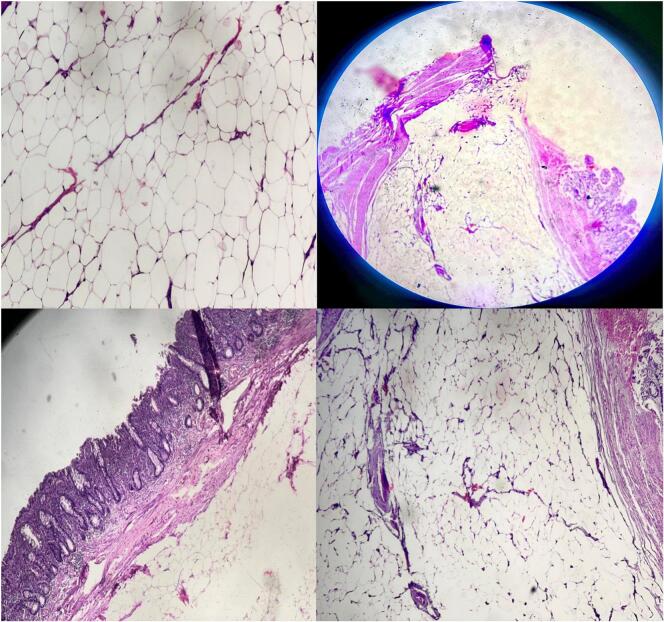
Pathological examination showing the resected lipoma with ulceration of the overlying mucosa.

The bowel segment proximal to the transition zone was distended, while the distal portion appeared to be collapsed. Upon performing enterotomy at the transition zone, a large pedunculated polyp, approximately 3 cm in size, was found to be causing a mechanical obstruction (Fig. 1). This polyp was confirmed to be an intramural lipoma, a rare cause of mechanical bowel obstruction in the absence of intussusception. Resection was performed (Fig. 2), and the bowel was repaired in two layers, with the abdominal wall closed. After surgery, the resected specimen was sent for pathological examination. The specimen, received in formalin, consisted of a brown-colored polypoid fragment of soft tissue measuring 3.5 × 2.5 × 2.5 cm. Upon cutting the specimen, it exhibited an appearance resembling adipose tissue. The base of the polyp measured 2/1 cm, while the tip measured 4/2 cm. The specimen was embedded at a 30 % rate (Fig. 3). Microscopic examination supported the diagnosis, confirming the final pathological diagnosis of Lipoma with ulceration of the overlying mucosa. The lesion was located in the distal ileum and was identified as a polypectomy specimen.

Forty-eight hours after surgery, the patient was started on a liquid diet, which was gradually advanced to a regular diet. His bowel function returned to normal, and he was discharged without complications.

## 3 Discussion

Small bowel lipomas are uncommon, benign growths of mature adipose tissue, most frequently located in the colon, but they can arise throughout the gastrointestinal tract, including the ileum. The ileum is one of the most common sites for tumors in the small intestine [8,9]. However, it is extremely rare to encounter intraluminal lipomas in the distal ileum that lead to mechanical obstruction, as seen in this case. A case analogous to ours presents a complete small intestine obstruction without intussusception due to a submucosal lipoma. In this instance, the lipoma was located in the jejunum [6]; whereas, in our case, it had been found in the distal ileum. To the best of our knowledge, this case report presents the first reported case of a small intestinal lipoma in the distal ileum leading to complete obstruction without intussusception.

Most gastrointestinal lipomas are asymptomatic and often go unnoticed until they become large enough to cause complications, such as partial or complete bowel obstruction. This obstruction usually occurs due to mechanisms like intussusception, but larger lipomas or those in specific locations can also lead to mechanical obstruction because of their size and/or location [10,11]. In this case, the lipoma caused a mechanical obstruction in the distal ileum without requiring intussusception, highlighting the variability in the mechanisms by which these benign lesions can lead to clinical complications.

Intraluminal lipomas, similar to other gastrointestinal lipomas, can cause abdominal pain and vomiting and may require surgical intervention in severe cases. However, in this case, the abdominal pain and vomiting were due to a non-ischemic mechanical obstruction. Bowel obstruction due to lipomas is rare and generally presents with complications such as intussusception or volvulus. Without these secondary issues, such mechanical obstruction often goes unnoticed or results in delayed diagnosis [12,13].

Before surgery, imaging plays a critical role in diagnosing lipomas, with X-ray often as an initial diagnostic tool. However, in this case, the abdominal X-ray showed signs of bowel obstruction but did not immediately reveal the presence of the lipoma, highlighting the challenges of diagnosing such lesions. While X-rays help detect bowel obstruction, advanced imaging techniques such as CT or MRI could provide a more detailed view, especially in identifying soft tissue lesions and distinguishing them from other differential diagnoses, such as tumors or mesenteric cysts [14,15].

Surgical removal remains the most effective treatment for symptomatic or obstructing lipomas. The lipoma was excised in this patient, and there were no complications in the subsequent bowel repair. Even though some report enucleation or laparoscopy, large or obstructive lipomas tend to be more favorably treated with laparotomy and en-bloc resection of the affected segment of the bowel [1]. This case illustrates that lipomas should also be considered an underlying cause of bowel obstruction, particularly in the setting of more common issues such as intussusception or volvulus.

## 4 Conclusion

This case highlights the rarity and clinical significance of intraluminal lipomas in the distal ileum as a cause of small bowel obstruction without associated intussusception. The final diagnosis was a submucosal intraluminal lipoma confirmed by histopathological examination. The patient was successfully treated with surgical resection via laparotomy, followed by primary bowel repair, leading to full recovery without complications. This case emphasizes the importance of including lipomas in the differential diagnosis of bowel obstruction and supports surgical resection as the definitive and effective management approach for symptomatic or obstructive intestinal lipomas.

## CRediT authorship contribution statement

Atabak Sedigh-namin: Data collection, data analysis, and manuscript writing, and first author.

Mehran Hatabi: Study design, data interpretation, and manuscript review, and first author.

Hamed Mohammadi Haris: Study concept and design, data interpretation, and manuscript review, and corresponding author.

Mohammadamin Vatankhah: Data interpretation.

Elmira Mousavi: Data interpretation.

Meisam Refaei: Data interpretation.

## Consent for publication

Written informed consent was obtained from the patient's legal guardian for publication of this case report and any accompanying images. A copy of the written consent is available for review by the Editor-in-Chief of this journal.

## Ethical approval

This study was conducted in accordance with the ethical principles outlined in the Research Ethics Committees of Ardabil University of Medical Sciences. As this research, it was determined to be exempt from formal ethical review by the Research Ethics Committees of Ardabil University of Medical Sciences.

## Funding

Not applicable.

## Declaration of competing interest

No conflicts of interest.

## Data Availability

If supporting data is needed contact the correspondence author.

## References

[1] Kerwan A., Al-Jabir A., Mathew G., Sohrabi C., Rashid R., Franchi T., Nicola M., Agha M., Agha R.A. (2025). Revised surgical CAse REport (SCARE) guideline: an update for the age of artificial intelligence. Premier Journal of Science.

[2] Pei M., Hu M., Chen W., Qin C. (2017). Multiple duodenal lipomas as a rare cause of upper gastrointestinal obstruction: case report and literature review. Gastroenterol. Res..

[3] Casiraghi T., Masetto A., Beltramo M. (2016). Intestinal obstruction caused by ileocolic and colocolic intussusception in an adult patient with cecal lipoma. Case Rep Surg.

[4] Goasguen N., Cattan P., Godiris-Petit G., Munoz-Bongrand N., Allez M., Lemann M., Sarfati E. (2008). Colonic lipoma: case report and literature review. (article in French). Gastroenterol. Clin. Biol..

[5] Rogy M., Mirza D., Berlakovich G., Winkelbauer F., Rauhs R. (1991). Submucous large-bowel lipomas--presentation and management. An 18-year study. Eur. J. Surg..

[6] Konik R.D., Rhodes R.A. (2018 Jul 2). Complete small bowel obstruction without intussusception due to a submucosal lipoma. J Surg Case Rep..

[7] Minaya Bravo A.M., Vera Mansilla C., Noguerales Fraguas F., Granell Vicent F.J. (2012). Ileocolic intussusception due to giant ileal lipoma: review of literature and report of a case. Int. J. Surg. Case Rep..

[8] Kakiuchi Y., Mashima H., Hori N., Takashima H. (Dec 2017). A small intestine volvulus caused by strangulation of a mesenteric lipoma: a case report. J. Med. Case Rep..

[9] Islam S., Hosein D., Dan D., Naraynsingh V. (19 sept 2016). Volvulus of ileum: a rare cause of small bowel obstruction. BMJ Case Rep..

[10] Cojocari N., David L. (4 avr 2020). Acute intestinal infarction due to diffuse jejunoileal and mesenteric lipomatosis in a 39-year-old woman [cit’e 26 nov 2021]. Am. J. Case Rep..

[11] Suga Y., Abdi E., Bekele M. (1 janv 1970). Giant mesenteric lipoma causing small bowel volvulus: a case report [cit’e 26 nov 2021]. Ethiop. J. Health Sci..

[12] Miranda J.A., Viana P.C.C., Meirelles L.R., Panizza P.S.B., Jureidini R., Horvat N. (2018). Torsion of a giant antimesenteric lipoma of the sigmoid: a rare cause of acute abdomen. Radiol. Case Rep..

[13] Buono G.D., Ricupati F., Amato G., Gulotta L., Romano G., Agrusa A. (2020). Small bowel volvulus due to a large intestinal lipoma: a rare case report. Int. J. Surg. Case Rep..

[14] Aydin H.N., Bertin P., Singh K., Arregui M. (août 2011). Safe techniques for endoscopic resection of gastrointestinal lipomas. Surg. Laparosc. Endosc. Percut. Tech..

[15] Cakmak G.K., Emre A.U., Tascilar O., Bektas S., Ucan B.D., Irkorucu O. (2007). Lipoma within inverted Meckel’s diverticulum as a cause of recurrent partial intestinal obstruction and hemorrhage: a case report and review of literature. World J. Gastroenterol..

